# Dual-Color Fluorescence Imaging to Monitor CYP3A4 and CYP3A7 Expression in Human Hepatic Carcinoma HepG2 and HepaRG Cells

**DOI:** 10.1371/journal.pone.0104123

**Published:** 2014-08-07

**Authors:** Saori Tsuji, Fumihiko Kawamura, Musashi Kubiura, Ayaka Hayashi, Tetsuya Ohbayashi, Yasuhiro Kazuki, Christophe Chesné, Mitsuo Oshimura, Masako Tada

**Affiliations:** 1 Bio Frontier Project Promotion Section, Organization for Tottori Industrial Promotion, Yonago, Japan; 2 Division of Molecular Genetics and Biofunction, Department of Biomedical Science, Institute of Regenerative Medicine and Biofunction, Graduate School of Medical Science, Tottori University, Yonago, Japan; 3 Division of Laboratory Animal Science, Research Center for Bioscience and Technology, Tottori University, Yonago, Japan; 4 Chromosome Engineering Research Center, Tottori University, Yonago, Japan; 5 Biopredic International, Rennes, France; Université de Technologie de Compiègne, France

## Abstract

Human adult hepatocytes expressing CYP3A4, a major cytochrome P450 enzyme, are required for cell-based assays to evaluate the potential risk of drug-drug interactions caused by transcriptional induction of P450 enzymes in early-phase drug discovery and development. However, CYP3A7 is preferentially expressed in premature hepatoblasts and major hepatic carcinoma cell lines. The human hepatocellular carcinoma cell line HepaRG possesses a high self-renewal capacity and can differentiate into hepatic cells similar to human adult hepatocytes *in vitro*. Transgenic HepaRG cells, in which the expression of fluorescent reporters is regulated by 35 kb regulatory elements of CYP3A4, have a distinct advantage over human hepatocytes isolated by collagenase perfusion, which are unstable in culture. Thus, we created transgenic HepaRG and HepG2 cells by replacing the protein-coding regions of human CYP3A4 and CYP3A7 with enhanced green fluorescent protein (EGFP) and DsRed reporters, respectively, in a bacterial artificial chromosome vector that included whole regulatory elements. The intensity of DsRed fluorescence was initially high during the proliferation of transgenic HepaRG cells. However, most EGFP-positive cells were derived from those in which DsRed fluorescence was extinguished. Comparative analyses in these transgenic clones showed that changes in the total fluorescence intensity of EGFP reflected fold changes in the mRNA level of endogenous CYP3A4. Moreover, CYP3A4 induction was monitored by the increase in EGFP fluorescence. Thus, this assay provides a real-time evaluation system for quality assurance of hepatic differentiation into CYP3A4-expressing cells, unfavourable CYP3A4 induction, and fluorescence-activated cell sorting-mediated enrichment of CYP3A4-expressing hepatocytes based on the total fluorescence intensities of fluorescent reporters, without the need for many time-consuming steps.

## Introduction

Any indication that a compound and/or one of its metabolites can induce the transcription of P450 (CYP) metabolic enzymes and hepatotoxicity is a serious problem in drug development. Such “CYP induction” may attenuate the pharmacological effect of the primary drug or those of subsequently administered drugs, effects that are known as drug-drug interactions. Thus, a system is needed to evaluate whether candidate compounds carry such risks. In addition to being cell-based, this assay should be inexpensive, easy to use, predictive, reproducible, mechanism-based, and applicable to high content screening (HCS). Such an assay requires cells that can be grown vigorously and quickly, that respond adequately to compounds, and, most importantly, that can be similar to human adult hepatocytes. In terms of the latter, CYP3A4 is an excellent marker of functional adult hepatocytes.

CYP3A4 is the predominant P450 enzyme and is responsible for the oxidation of 50–60% of clinical drugs [1]. CYP3A enzymes are subject to multiple levels of regulation, as follows: (1) tissue-restricted expression, (2) cell type-specific expression, (3) developmental regulation, (4) xenobiotic induction, and (5) reactivation of fetal-type CYP3A7 through hepatoblast expansion. CYP3A4 is specifically expressed in the liver, small intestine, and colon. In the liver, CYP3A expression is mostly restricted to hepatocytes surrounding the central veins within zone 3. Some CYP3A genes are developmentally regulated. The major CYP3A isoform expressed in human fetal liver is CYP3A7, which changes to CYP3A4 during the first week of postnatal life [2], [3]. A similar developmental switch from Cyp3a16 to Cyp3a11 has been observed in mice [4], [5]. Xenobiotics induce CYP3A4 when they bind to the pregnane X receptor (PXR) and subsequently form a heterodimer with retinoid X receptor (RXR). This complex binds to the predominant cis-acting elements responsible for xenobiotic induction located within 8 kb upstream of the transcription initiation site termed XREM (dNR1 and dNR2) and proximal pNR [6], [7], [8], resulting in the accumulation of metabolites.

CYP3A4 and CYP3A7 share more than 90% homology within their promoter regions and protein-coding regions [9]. However, CYP3A7 differs from CYP3A4 in terms of its capacity to perform enzymatic reactions as well as its substrate specificity. Moreover, PXR is not detected in human fetuses, and CYP3A7 is mainly induced through a homodimer of glucocorticoid receptor [10]. Thus, CYP3A4-expressing adult-type hepatocytes are required for drug toxicity tests. However, the DNA sequences of CYP3A4 and CYP3A7 are highly similar, which makes it difficult to identify CYP3A4-expressing mature hepatocytes or CYP3A7-expressing premature hepatoblastic cells by performing immunostaining.

Transgenic mice carrying multi-copies of a 13 kb fragment of the 5′-region of CYP3A4 linked to β-galactosidase (β-gal) or luciferase (luc) have been reported [11], [12], [13]. The β-gal reporter of CYP3A4 is specifically expressed in zone 3 of the liver in adult mice, and both reporter genes respond to representative CYP3A4 inducers *in vivo*. Thus, we predicted that fluorescent reporters could be used to generate a sensitive and accurate monitoring system of CYP3A4 expression in real time. Moreover, CYP3A7 and α-fetoprotein (AFP) can be used as hepatoblastic markers [14], [15]. Thus, a fluorescent reporter whose expression is under the control of the enhancer and promoter regions of CYP3A7 will help to monitor hepatoblastic expansion in culture. We therefore anticipated that the enhanced green fluorescent protein (EGFP) and the red fluorescent reporter gene (DsRed) could be used to distinguish between CYP3A4 and CYP3A7, respectively. Moreover, transcriptional regulation of CYP3A genes appears to be species specific. Therefore, *in vitro* assays to predict the level to which a given compound induces CYP3A4 expression should be devised using human adult-type hepatocytes that retain the metabolic activities of adult human hepatocytes.

The human hepatoma cell line HepaRG cells have great plasticity and can differentiate into human adult hepatocyte-like and cholangiocyte-like cells *in vitro* when cultured in the presence of corticoids and dimethylsulfoxide (DMSO) [16], [17]. Thus, we used HepaRG cells as well as the human hepatoblastoma cell line HepG2. First, the open reading frames (ORFs) of CYP3A4 and CYP3A7 were replaced with EGFP and DsRed, respectively, in a bacterial artificial chromosome (BAC) vector (4G/7R BAC). All the BAC transgenic HepaRG cells initially exhibited strong DsRed fluorescence; however, this fluorescence was extinguished immediately after differentiation culturing and EGFP fluorescence increased a few days later. Thus, the intensity of EGFP fluorescence can be used as a quality-control measure to quantify CYP3A4-expressing functional hepatocytes. Moreover, quantitative RT-PCR (qRT-PCR) analyses showed that changes in the total fluorescence intensity of EGFP reflected those in the endogenous mRNA level of CYP3A4 in HepG2 and HepaRG transgenic clones. Thus, these transgenic cells reduce the time and costs required to estimate the mRNA or protein level of CYP3A4. Moreover, EGFP-positive transgenic HepaRG cells can be used as an alternative to human adult-type hepatocytes for various analyses of drug metabolism, drug-drug interactions, hepatic toxicity, and the carcinogenicities of foreign chemicals.

## Results

### The 4G/7R BAC and transgenic HepG2 and HepaRG cells

**Figure 1:**
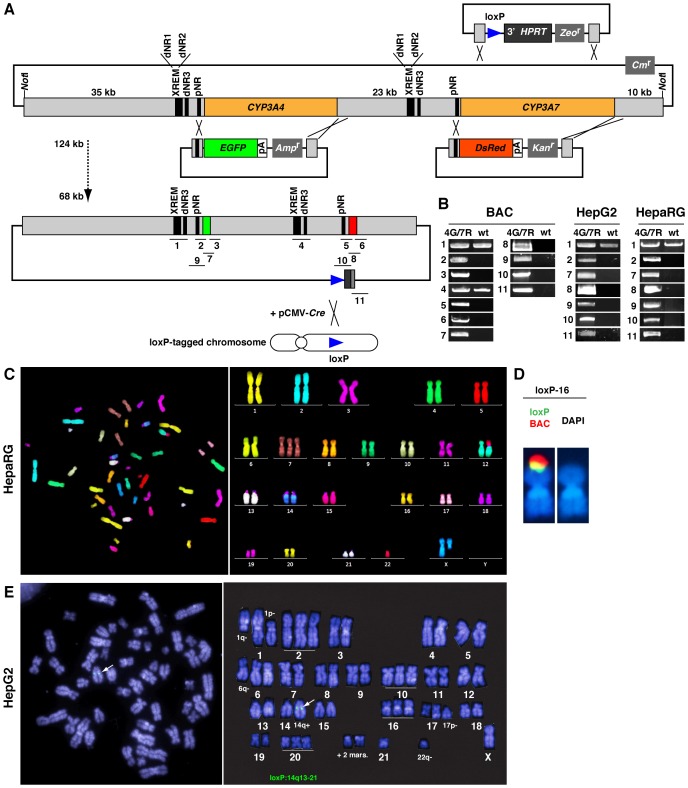
The CYP3A4G/7R (4G/7R) BAC and transgenic HepG2 and HepaRG cells. (A) Strategy of RP11-757A13 BAC recombineering using three knock-in plasmid vectors (top) and the resultant 4G/7R BAC (below). Orange, ORFs of CYP3A4/7; green, EGFP; red, DsRed; blue triangles, loxP sites. Numbers (1–11) beneath the 4G/7R BAC indicate regions analyzed by genomic PCR. (B) Genomic PCR analyses using the primer sets shown in A. DNA was isolated from the BAC, HepG2 cells, and HepaRG cells before (WT) and after genetic manipulation. (C) mFISH analysis shows that transgenic HepaRG clone 3 cells have the same karyotype as the parental HepaRG cells, except for a long-arm deletion of the X chromosome. (D) FISH mapping shows that the 4G/7R BAC has been introduced into the acceptor loxP site on human chromosome 16 in transgenic HepaRG cells. (E) FISH mapping in loxP-bearing HepG2 cells shows that a loxP site has been created in a region proximal to human chromosome 14. Arrows indicate the loxP site.

CYP3A4 and CYP3A7 (CYP3A4/7) are located adjacent to each other on human chromosome 7. The RP11-757A13 clone was chosen from BAC libraries. Sequence information was obtained from the NCBI and the accession numbers were as follows: RP11-757A13, AC069294; CYP3A4 mRNA, NM_017460; and CYP3A7 mRNA, NM_000765. In this BAC clone, the 123 kb NotI-digested DNA fragment of CYP3A4/7 had been inserted into the EcoRI site of the 11.5 kb pBACe3.6. The wild-type (WT) BAC was introduced into DY380 E. coli, and chloramphenicol-resistant (Cm^r^) transformants were selected. The CYP3A4/7 genomic regions were extensively analyzed by PCR to ascertain the maintenance of major transcriptional regulatory elements. First, three knock-in vectors were constructed for BAC recombineering (Fig. 1A). To introduce a single BAC clone into a specific acceptor site in the host cells using Cre, a loxP site was introduced into the recombinant BAC and into the genome of the host cells. Zeocin-resistant (Zeo^r^) loxP-bearing BAC clones were selected. Second, the ORF of CYP3A4 was replaced with EGFP, and ampicillin-resistant (Amp^r^)/Zeo^r^ clones were selected. Third, the ORF of CYP3A7 was replaced with DsRed, and kanamycin-resistant (Kan^r^)/Amp^r^/Zeo^r^ clones were selected. Clones with the 4G/7R BAC were selected by genomic PCR using numerous primer sets, which are shown in Table 1 (Figs. 1A and B). Direct sequencing showed that the important regulatory elements dNR1, dNR2, dNR3, and pNR of the CYP3A4 and CYP3A7 genes were all intact in the modified BAC vector (Fig. 1A and Table 2).

**Table 1:** PCR primers used for genomic RCR analyses.

Sets	Positions	Size	Primers	Sequences (5′ to 3′)
1	dNR1-dNR2-dNR3 (-7901 to -7316 nt of CYP3A4)	585 bp	A4XREM-F	CATTTGATTATCAAAGAAACTC
			A4XREM-R	GAATGGTTATAAGATCATCTCAATG
2	pNR (-7901 nt to the first ATG codon of CYP3A4) and 5′ EGFP)	957 bp	CYP3A4-2F	CAACAGAATCACAGAGGACCAGC
			5′ EGFP-R	CAGCTCCTCGCCCTTGCTCAC
3	3′ EGFP to +29836 nt of CYP3A4	4178 bp	Amp^R^-F	GTAGAAAAGATCAAAGGATC
			CYP3A4-4R	TTAGCTGGGGTGAGATGCTATCTCAC
4	dNR1-dNR2-dNR3 (-7895 to -7304 nt) of CYP3A7	593 bp	A7XREM-F	CCAATAATCAATGAAACTCATG
			A7XREM-R	ATGATCTCATCAACAGATTA
5	pNR (-865 nt to the first ATG codon of CYP3A7) and 5′ DsRed	929 bp	CYP3A7-2F	GCTGTATTAATGACCTAAGAAGATGGAGTG
			5′ DsRed-R	GATGACGTCCTCGGAGGAGGC
6	3′ DsRed to +32935 nt of CYP3A7	3690 bp	Kan/Neo^R^-F	GCCTTCTTGACGAGTTCTTC
			CYP3A7-4R	GGCTCACTGCCACCTATGCCTCATG
7	EGFP	720 bp	EGFP ORF-F	ATGGTGAGCAAGGGCGAGGA
			EGFP ORF-R	TTACTTGTACAGCTCGTCCATGC
8	DsRed	647 bp	DsRed ORF-F	GCCTCCTCCGAGGACGTCATC
			DsRed ORF-R	GCGCGCTCGTACTGCTCCAC
9	-3000 nt to the first ATG codon of CYP3A4 and 5′ EGFP	3024 bp	CYP3A4-1F	CAAAGTTGACCAAGACCAACTTTGGTTG
			5′ EGFP-R	CAGCTCCTCGCCCTTGCTCAC
10	-5598 nt to the first ATG codon of CYP3A7 and 5′ DsRed	5662 bp	CYP3A7-1F	CTTGGAATCCCAGCAAGAACACCACTGATG
			5′ DsRed-R	GATGACGTCCTCGGAGGAGGC
11	3′ loxP to BAC	1.2 kb	loxP 1650-F	CCGAAAGGCCCAACAGGCAAGCTGATGAGA
			BAC e3.6 5839-AS	AACTTCTGTGCTTAAAACGTCATCTGCATC

**Table 2:** Promoter elements maintained in the CYP3A4G/7R BAC clone.

Elements	Size	Genes	Sequences (5′ to 3′)	Positions (nt)
dNR1 (DR-3)	15 bp	CYP3A4	TGAACTtgcTGACCC	−7822 to −7808
		CYP3A7	TGAACTtgcTGACCC	−7819 to −7805
dNR2 (ER-6)	18 bp	CYP3A4	TGAAATcatgtcGGTTCA	−7778 to −7761
		CYP3A7	TGAAATcatgtcAGTTCA	−7775 to −7758
dNR3 (DR-3)	15 bp	CYP3A4	TATTGTtatTGAACT	−7376 to −7362
		CYP3A7	TATTATtatTGAACT	−7389 to −7375
pNR (ER-6)	18 bp	CYP3A4	TGAACTcaaaggAGGTCA	−272 to −255
		CYP3A7	TTAACTcaatggAGGTCA	−271 to −254

Nucleotides that differ between CYP3A4 and CYP3A7 are underlined.

Transcriptional regulation of CYP3A genes appears to be species specific [18], [19], [20]. Thus, the ultimate goal of the *in vitro* assays was to predict the level to which CYP3A4 expression is induced in human cells. First, we isolated several transgenic HepaRG and HepG2 clones that were hygromycin-resistant (Hyg^r^) and therefore had a loxP site. Transgenic cells with the 4G/7R BAC were then created using Cre-mediated recombination, and G418-resistant (G418^r^) clones were selected. We mainly used the transgenic HepaRG clone 3, in which the 4G/7R BAC was inserted into a loxP site on human chromosome 16. Multi-color fluorescence *in situ* hybridisation (mFISH) analysis showed that the karyotype of these cells was similar to that of the parental HepaRG cells [16] (Fig. 1C). The insertion of the 4G/7R BAC into a loxP site on human chromosome 16 in these cells was confirmed using dual-color fluorescence *in situ* hybridization (FISH) mapping analysis (Fig. 1D). A loxP site was created on human chromosome 14 in HepG2 cells, and these cells were used to generate several 4G/7R BAC transgenic HepG2 clones, of which clone 87 was mainly used (Fig. 1E). Genomic PCR using the primer sets shown in Table 1 confirmed that the major elements in the 4G/7R BAC were retained in these transgenic cells (Fig. 1B, right).

### Rifampicin (RIF) increases the mRNA and protein levels of EGFP in transgenic HepG2 cells

**Figure 2:**
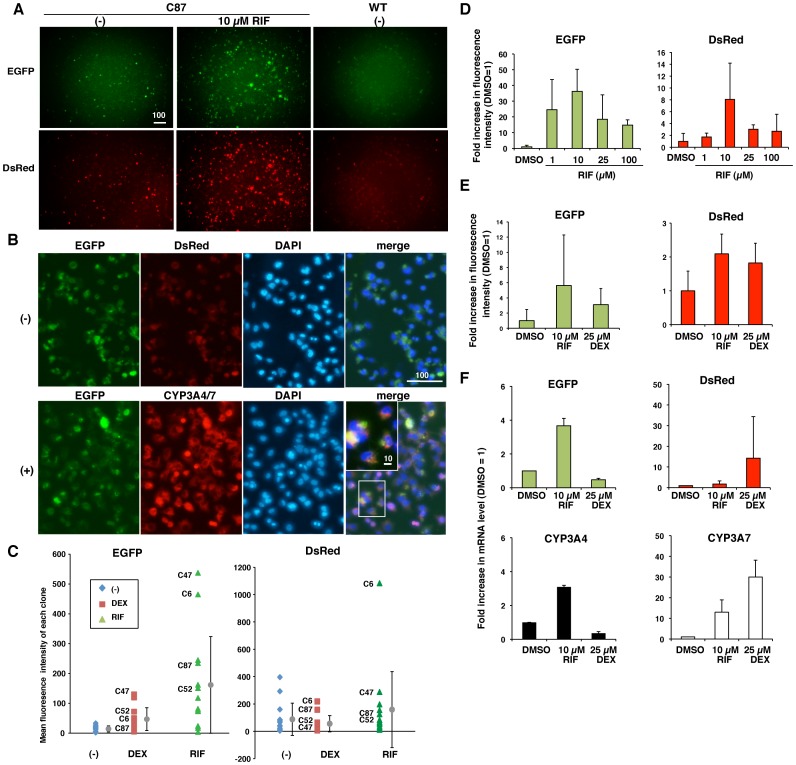
RIF increases the number of EGFP- and/or DsRed-positive transgenic HepG2 cells, as well as the fluorescence intensities and mRNA levels of EGFP and DsRed. (A) EGFP fluorescence and DsRed fluorescence of transgenic HepG2 clone C87 cells before (−) and after treatment with 10 µM RIF. WT: non-transgenic wild-type HepG2 cells. (B) Immunostaining of HepG2 cells for CYP3A4/7. HepG2 C87 cells exhibit EGFP and DsRed fluorescence even after paraformaldehyde fixation (−). Signals for CYP3A4/7 were much brighter when an Alexa546-conjugated secondary antibody was used (+). The scale bar indicates 100 µm in the image and 10 µm in the inset. (C) Representative transgenic HepG2 clones were selected in which 200 µM DEX and 100 µM RIF treatment induced high levels of fluorescence. Each point displays the mean fluorescence intensity of three independent areas in a well. The error bars show the mean ± standard deviation (SD) of the fluorescence intensity. (D) HepG2 C87 cells at passage 7 show simultaneous induction of EGFP and DsRed fluorescence when treated with 1–100 µM RIF. For each treatment, three wells were analyzed, and the mean fluorescence intensity of three independent areas in each well was calculated, and the fold increase in fluorescence intensity in comparison to the level in 0.1% DMSO-treated cells (set at 1) is shown. (E) Fluorescence microscopic analyses showing simultaneous induction of EGFP and DsRed fluorescence following treatment with 10 µM RIF or 25 µM DEX in HepG2 C87 cells at passage 7 and passage 10. Cells were cultured in three wells for each treatment. Fluorescence intensities were measured in three areas for each well. The mean ± SD of the fold increase in fluorescence intensity relative to the level in 0.1% DMSO-treated cells (set at 1) is shown. (F) qRT-PCR analyses showing that the fold increases in EGFP and DsRed mRNA levels matched well with those in endogenous CYP3A4 (left graphs) and CYP3A7 (right graphs) mRNA, respectively. The analyses were performed using the same HepG2 C87 cell samples as in the microscopic analyses (Fig. 2E). The mean ± SD of the fold increase in the mRNA level relative to the level in 0.1% DMSO-treated cells (set at 1) is shown.

HepG2 cells have many characteristics that are similar to premature hepatic cells, including a low level of CYP3A4/7 expression and weak responses to CYP3A4 inducers [21]. However, PXR expression is higher in HepG2 cells than in human fetal hepatic cells [10]. We tested the response of transgenic HepG2 cells to the antibiotic RIF, which can induce CYP3A4 expression. In transgenic C87 HepG2 cells, EGFP and DsRed were weakly detected when cells were cultured under normal conditions. However, the fluorescence intensities of EGFP and DsRed, as well as the number of cells positive for these markers, increased significantly following treatment with 10 µM RIF for 48 h (Fig. 2A). Direct comparison of EGFP expression and endogenous CYP3A4 immunolocalization is informative. However, there are some technical issues: (1) CYP3A4 and CYP3A7 cannot be distinguished from each other using antibodies because the two proteins are highly similar, and (2) every transgenic cell exhibits red and green fluorescence at varying levels. Following immunostaining with a combination of primary anti-CYP3A4/7 antibody and red fluorescent marker-conjugated secondary antibody, red fluorescence was more intense in immunostained C87 cells (Fig. 2B, (+), CYP3A4/7) than in untreated C87 cells (Fig. 2B, (-), DsRed). Moreover, EGFP positive cells were strongly labeled by the anti-CYP3A4/7 antibody (Fig. 2B, (+), EGFP). Thus, EGFP-positive cells are likely to represent CYP3A4- and/or CYP3A7-expressing cells. On the basis of total fluorescence intensity, we selected clones that were sensitive to 100 µM RIF and 200 µM dexamethasone (DEX), another CYP3A inducer. For each clone, three wells were analyzed, and three areas were imaged in each well. The mean and standard deviation (SD) of the fluorescence intensities of EGFP and DsRed were then calculated for each clone (Fig. 2C). Of 17 clones, C87 was selected for further analyses because EGFP fluorescence intensity in these cells had a high signal-to-noise ratio.

In this study, we cultured transgenic HepG2 and HepaRG cells under comparable conditions and treated three wells of cells with DMSO, RIF, or DEX. Each well was independently analyzed in triplicate by fluorescence microscopy, qRT-PCR, Western blotting, or fluorescence-activated cell sorting (FACS). First, the mean values of triplicated measures were calculated for each well. Second, these values were divided by the corresponding mean values of β-actin to normalize for variation in the number of cells per well. Third, the fold increase or relative value of this normalized mean value was obtained by comparison to the appropriate reference sample, i.e., DMSO-treated cells, adult liver (AL), fetal liver (FL), or non-transgenic wild-type (WT) cells. Cells treated with 0.1% DMSO were used as the control because this was the solvent in which the CYP inducers were dissolved. Finally, the mean values and standard deviations (SD) of these relative values were calculated for 3–9 wells. These relative values were statistically analyzed using the Mann-Whitney U-test.

Remarkably, EGFP and DsRed fluorescence intensities responded similarly to 1–100 µM RIF treatment in HepG2 C87 cells. The EGFP and DsRed fluorescence intensities were 36.2±14.1-fold and 8.1±6.1-fold higher in 10 µM RIF-treated cells than in DMSO-treated control cells (p<0.05), respectively (Fig. 2D). To determine whether changes in the fluorescence intensities of EGFP and DsRed reflect those in the levels of endogenous CYP3A4 and CYP3A7, respectively, we performed fluorometric analyses followed by qRT-PCR analyses. To analyze the effects of treatment with 10 µM RIF and 25 µM DEX, we used HepG2 C87 cells from two passages, namely, around passage 7 (early) and passage 10 (mid), and treated these cells with RIF, DEX, or DMSO for 48 h. Then, three areas per well were imaged by fluorescence microscopy (Fig. 2E). The cells were also used for qRT-PCR analyses of the mRNA levels of CYP3A4, CYP3A7, EGFP, and DsRed (Fig. 2F). Then, the relative mRNA levels of CYP3A4 and CYP3A7 in these cells were calculated with the expression level in 0.1% DMSO-treated cells set at 1. The mean ± SD values are shown in Figure 2F. Treatment with 10 µM RIF similarly enhanced EGFP fluorescence and mRNA levels of EGFP and CYP3A4 (Figs. 2E and F). EGFP fluorescence intensity was 5.6±3.1-fold higher in 10 µM RIF-treated C87 cells than in DMSO-treated control cells (p<0.1) (Fig. 2E, left). The mRNA levels of EGFP and CYP3A4 were 3.7±0.4-fold and 3.1±0.1-fold higher in RIF-treated C87 cells than in DMSO-treated control cells, respectively (p<0.05) (Fig. 2F, left graphs). DsRed fluorescence intensity was 2-fold higher in cells treated with 10 µM RIF or 25 µM DEX than in DMSO-treated control cells (p<0.1) (Fig. 2E, right). Treatment with 25 µM DEX significantly enhanced DsRed and CYP3A7 mRNA expression (p<0.05) (Fig. 2F, right graphs). Treatment with 10 µM RIF significantly induced mRNA expression of CYP3A7 (p<0.05), but increased mRNA expression of DsRed less (Fig. 2F, right graphs). In contrast to EGFP, there was a discrepancy between the DsRed fluorescence intensity and the mRNA levels of DsRed and endogenous CYP3A7. To visualize DsRed fluorescence, the mRNA level of DsRed must reach a threshold level in each cell. Treatment with 25 µM DEX may weakly induce mRNA expression of CYP3A7 and DsRed in C87 cells, but this might be insufficient to microscopically detect DsRed fluorescence in each cell.

### CYP3A inducers effectively increase the number of EGFP-positive HepG2 cells

**Figure 3:**
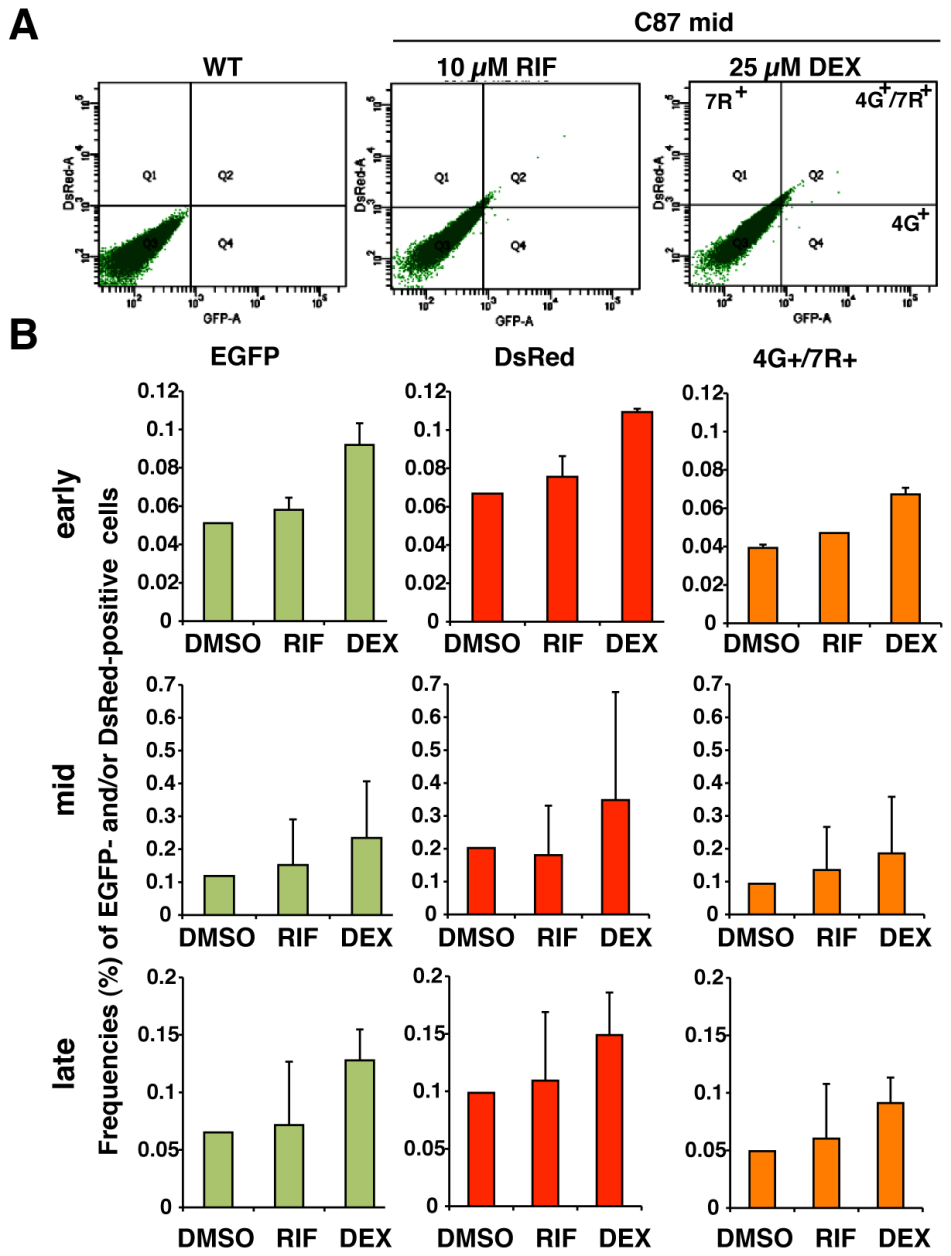
CYP3A inducers markedly increase the number of EGFP-positive transgenic HepG2 cells. (A) FACS analyses showing the remarkable increase in EGFP-positive cells in the transgenic HepG2 C87 cell population in comparison to the HepG2 WT cell population, and the appearance of cells double-positive for EGFP and DsRed (4G^+^/7R^+^) following treatment with 10 µM RIF or 25 µM DEX. (B) Frequencies (%) of EGFP-, DsRed-, and double-positive (4G^+^/7R^+^) cells among the total number of living cells are shown for HepG2 C87 cells at three passages: early (passage 7), mid (passage 10), and late (passage 14).

FACS analyses of transgenic HepG2 C87 cells were performed at three different passages, namely, around passage 7 (early), passage 10 (mid), and around P14 (late). Cells were cultured under comparable conditions, and then three wells were treated with DMSO, 10 µM RIF or 25 µM DEX. The frequencies (%) of EGFP-positive cells and/or DsRed-positive cells were analyzed among approximately 50,000 living cells per sample. Treatment with 25 µM DEX significantly increased the number of EGFP-positive cells and/or DsRed-positive cells (n = 9, p<0.05) (Figs. 3A and B). Treatment with 10 µM RIF also increased the number of EGFP- and/or DsRed-positive cells, although this increase was not significant (Figs. 3A and B). Most DsRed-positive cells were also EGFP-positive (Fig. 3A, 4G ^+^/7R^+^). RIF can co-induce CYP3A4 and CYP3A7 in adult human hepatocytes in culture [22]. However, it is unclear whether CYP3A4 and CYP3A7 located adjacent to each other on a chromosome are activated in the different cells, in the same cell, or on the same DNA strand. The co-expression of EGFP and DsRed from a single copy of the 4G/7R BAC in the same cell indicates two possibilities, as follows: (1) CYP3A4 inducers bind to the regulatory elements of CYP3A4 and CYP3A7 on the same DNA strand at same time, or (2) CYP3A4 inducers bind only to the regulatory elements of CYP3A4 and indirectly activate the neighbouring promoter region of CYP3A7 owing to chromatin opening on the same DNA strand.

**Figure 4:**
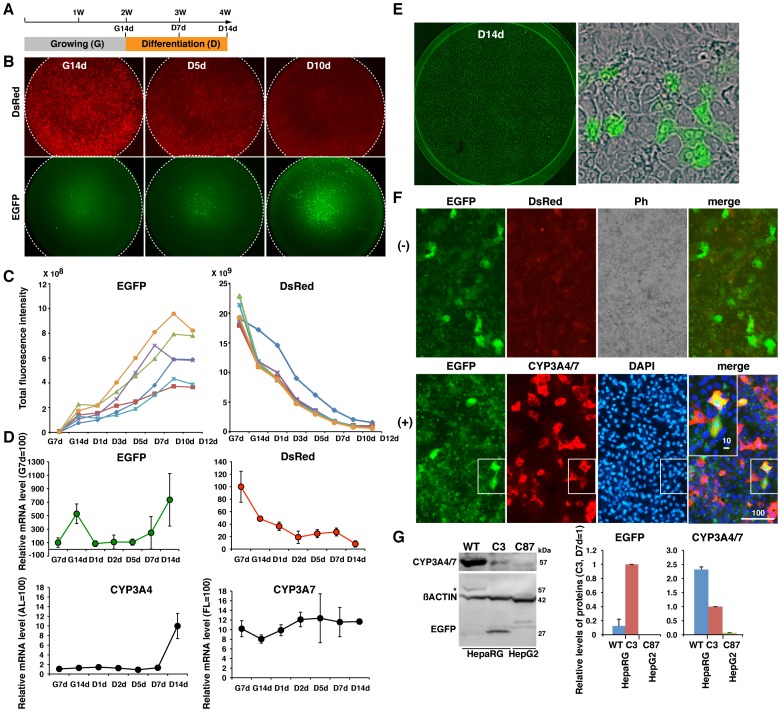
Real-time monitoring of CYP3A4 expression in differentiated transgenic HepaRG cells according to EGFP fluorescence. (A) Strategy for hepatic differentiation of HepaRG cells *in vitro*. (B) Images of DsRed and EGFP in transgenic HepaRG clone 3 cells cultured in a 96-well plate at G14d, and after 5 days (D5d) and 10 days (D10d) of differentiation culturing. (C) Total fluorescence intensities of EGFP or DsRed in each of 6 wells during the culture of cells that have undergone 7 days of growing (G7d) until D12d. The different colored lines denote different wells. (D) qRT-PCR analyses showing the mRNA levels of EGFP, DsRed, endogenous CYP3A4, and endogenous CYP3A7 in HepaRG cells from G7d to D12d. The mean ± SD of three independent cultures were calculated, and the mRNA levels in cells relative to those in AL or FL (set at 100) are shown. (E) The morphology of EGFP-positive HepaRG cells at D14d is distinct from that of the other large flat cells. (F) Immunostaining of HepaRG C3 cells for anti-CYP3A4/7 antibody at D14d. (-) and (+) show cells before and after immunostaining, respectively. The scale bar is 100 µm in the image and 10 µm in the inset. Ph: phase-contrast images. (G) Western blot analysis of CYP3A4/7 and EGFP in HepaRG WT cells, HepaRG C3 cells, and HepG2 C87 cells. Signal intensities are shown relative to those in HepaRG C3 cells at D7d (set at 1).

### Distinctive characteristics of CYP3A4-expressing differentiated HepaRG cells

HepaRG cells possess various functions that are similar to those of mature hepatocytes [16], [17], [23]. HepaRG cells actively self-renew under appropriate culture conditions; however, some cells begin to trans-differentiate into bipotent hepatic progenitors [23] and acquire morphological and functional characteristics typical of adult human hepatocytes when cultured for 14 days in differentiation-inducing conditions (D14d) [23] (Fig. S1A). Immunostaining showed that WT HepaRG cells at D14d were positive for albumin (ALB), cytokeratin (CK) 8/18, and alpha-1-antitrypsin (AAT), and included biliary-like cells that were positive for CK19. The differentiated HepaRG cells could be subdivided into two subtypes, namely, one which exhibited reactivity to an antibody against both CYP3A4 and CYP3A7, and another which did not (Fig. S1B). qRT-PCR analyses were performed using cells at various stages of differentiation, from immediately after the 14-day growing stage (G14d) to D15d. Expression of CYP3A4 was up to 1.8-fold higher in cells that had been cultured for 7 days in differentiation-inducing conditions (D7d) than in human adult liver (Fig. S1C, upper graph). By contrast, at the end of G14d, CYP3A7 expression was a few percent of the level in human fetal liver (Fig. S1C, lower graph); thus, HepaRG cells that exhibit reactivity to the anti-CYP3A4/7 antibody are likely to be CYP3A4-expressing cells. However, it is uncertain how the fate of CYP3A4-expressing cells is regulated. This information will be useful to obtain functional stem cell-derived adult-type hepatocytes that possess a high drug-metabolising ability. Moreover, the response of WT HepaRG cells to treatment with 10 µM RIF or 25 µM DEX was analyzed at D14d (n = 4). The mRNA level of CYP3A4 was increased 4.9±3.5-fold and 3.6±1.9-fold in the cells treated with RIF or DEX, respectively (p <0.05) (Fig. S1D). Thus, we expected that transgenic HepaRG cells bearing the 4G/7R BAC would provide a system to easily monitor CYP3A4 expression and transcriptional induction of CYP3A4 on the basis of EGFP fluorescence intensity.

### Identification of mature hepatocytes that have differentiated from transgenic HepaRG cells, according to EGFP fluorescence

All transgenic HepaRG cells exhibited strong DsRed fluorescence during the growing culture period, showing that undifferentiated HepaRG cells express CYP3A7 (Fig. 4A and B, G14d). HepaRG clone 3 cells were differentiated, and the total fluorescence intensity in each of six wells of a 96-well culture plate was measured from G7d until day 12 of differentiation culturing (D12d) using the LEAP imaging system. Over this period, EGFP fluorescence intensity increased (p<0.05), whereas DsRed fluorescence intensity was gradually extinguished in all the wells (p<0.05) (Figs. 4B and C). qRT-PCR analyses were performed in transgenic HepaRG C3 cells at three different passages around passage 14. mRNA expression of EGFP was up to 7-fold higher at D14d than at G7d (p<0.05), while mRNA expression of DsRed at D14d was 8% of that at G7d (p<0.05) (Fig. 4D, upper graph). mRNA expression of endogenous CYP3A4 was significantly increased during differentiation culturing from G7d to D14d, and reached up to 10% of that in human AL (p<0.05). The mRNA levels of EGFP and CYP3A4 correlated with each other. mRNA expression of DsRed gradually decreased during differentiation culturing from G7d to D14d, similar to DsRed fluorescence (p<0.05). By contrast, mRNA expression of endogenous CYP3A7 did not correlate with that of DsRed and only slightly changed during differentiation (Fig. 4D, lower graph). Each 4G/7R BAC transgenic cell carries a single copy of the DsRed reporter gene. Thus, high levels of DsRed mRNA transcription may be required to visualize DsRed fluorescence in each cell. Maintenance of CYP3A7 expression throughout the differentiation period, as seen in Figure 4D, indicates that the differentiated cell population contained non-fluorescent cells that expressed CYP3A7 mRNA at low levels. This might be particularly pronounced in HepaRG C3 cells with a reduced capacity to differentiate toward CYP3A4-expressing cells, although CYP3A7 expression was detected in differentiated WT HepaRG cells (Fig. S1C). The capacities of HepaRG C3 cells and WT HepaRG cells to differentiate were compared by Western blotting, as described below.

HepaRG C3 cells grown on ordinary culture plates were highly and uniformly EGFP-positive, and EGFP-positive cells often formed colonies of mononuclear small cells (Fig. 4E). DsRed fluorescence was already reduced in HepaRG C3 cells by D14d, and no cells exhibited strong red fluorescence (Fig. 4F, (−), DsRed). However, following immunostaining with an anti-CYP3A4/7 primary antibody and a red fluorescent marker-conjugated secondary antibody, strong red fluorescence was detected in HepaRG C3 cells (Fig. 4F, (+), CYP3A4/7). Moreover, EGFP-positive cells were strongly labeled by the anti-CYP3A4/7 antibody (Fig. 4F, (+), merge). Thus, EGFP-positive HepaRG cells likely express CYP3A4.

Next, by performing Western blotting using 10 µg of total protein in triplicate, the protein levels of CYP3A4/7 (57 kDa) were normalized against that of β-actin (42 kDa), and compared between WT and C3 HepaRG cells at D7d (Fig. 4G). CYP3A4/7 expression was 2-fold higher in HepaRG WT cells than in HepaRG C3 cells (p<0.05). The EGFP band at 27 kDa was detected in HepaRG C3 cells, but not in HepaRG WT cells. EGFP was not detected when 20 µg of total protein from HepG2 C87 cells was subjected to Western blotting, showing that EGFP expression was much higher in HepaRG C3 cells than in HepG2 C87 cells.

### FACS-mediated isolation of mature hepatocytes from differentiated transgenic HepaRG cells, according to EGFP fluorescence

**Figure 5:**
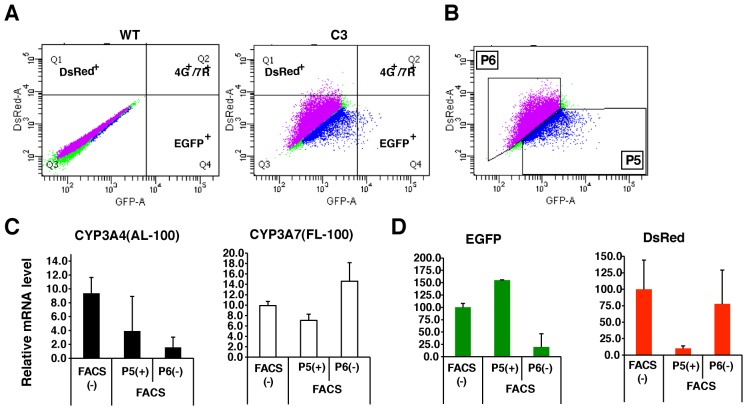
FACS-mediated isolation of CYP3A4-expressing hepatocytes from differentiated transgenic HepaRG cells according to EGFP fluorescence. (A) FACS analyses showing the remarkable increase in EGFP-positive cells or DsRed-positive cells in the transgenic HepaRG C3 cell population in comparison to the HepaRG WT cell population. Cells double-positive for EGFP and DsRed (4G^+^/7R^+^) were not detected. (B) Cells were separated into two fractions: P5(+), a fraction enriched with EGFP-positive cells and P6(−), a fraction enriched with DsRed-positive cells. (C) Relative mRNA levels of CYP3A4 (AL = 100) and CYP3A7 (FL = 100) in sorted P5(+), sorted P6(−) cells, and unsorted cells (FACS(−)). Graphs show the mean ± SD (n = 3). (D) Relative mRNA levels of EGFP and DsRed in sorted P5(+) and P6(−) cells (levels in unsorted cells (FACS(−)) were set at 100). Graphs show the mean ± SD (n = 3).

Three comparative cultures of transgenic C3 and WT HepaRG cells at D5d were prepared, and then FACS analyses were performed in triplicate for each sample. Various amounts of DsRed- or EGFP-expressing cells were detected, but 4G^+^/7R^+^ double-positive cells were rarely detected (Fig. 5A). The cell population at D5d also contained a number of non-fluorescent cells, which comprised hepatic cells in which CYP3A7 mRNA expression was weak or absent as well as biliary-like cells. Cells were separated into two fractions by FACS; a fraction enriched with EGFP-positive cells (Fig. 5B, P5(+)) and a fraction containing EGFP-negative cells (Fig. 5B, P6(−)), although contamination of either fraction with non-fluorescent cells could not be avoided. RNA was prepared from these sorted cells and used for qRT-PCR analyses. Unsorted cells were used to monitor cell damage caused by FACS analysis (Fig. 5C and D, FACS(−)). The mRNA level of CYP3A4 was lower in P5(+) than in unsorted cells, but was higher in P5(+) than in P6(−) (p<0.1) (Fig. 5C, left). Moreover, the mRNA level of CYP3A7 was lower in P5(+) than in P6(−) (p<0.05), showing that non-fluorescent cells in P5(+) likely express low levels of CYP3A7 (Fig. 5C, right). Remarkably, FACS enriched EGFP-positive cells in the P5(+) fraction, while DsRed-positive cells were preferentially isolated in the P6(−) fraction (p<0.05) (Fig. 5D). Thus, after technical improvement of the FACS procedure and enrichment of EGFP-positive differentiated HepaRG cells, EGFP-based FACS is technically viable to enrich CYP3A4-expressing hepatocytes.

### Recovery of frozen EGFP-positive HepaRG cells

**Figure 6:**
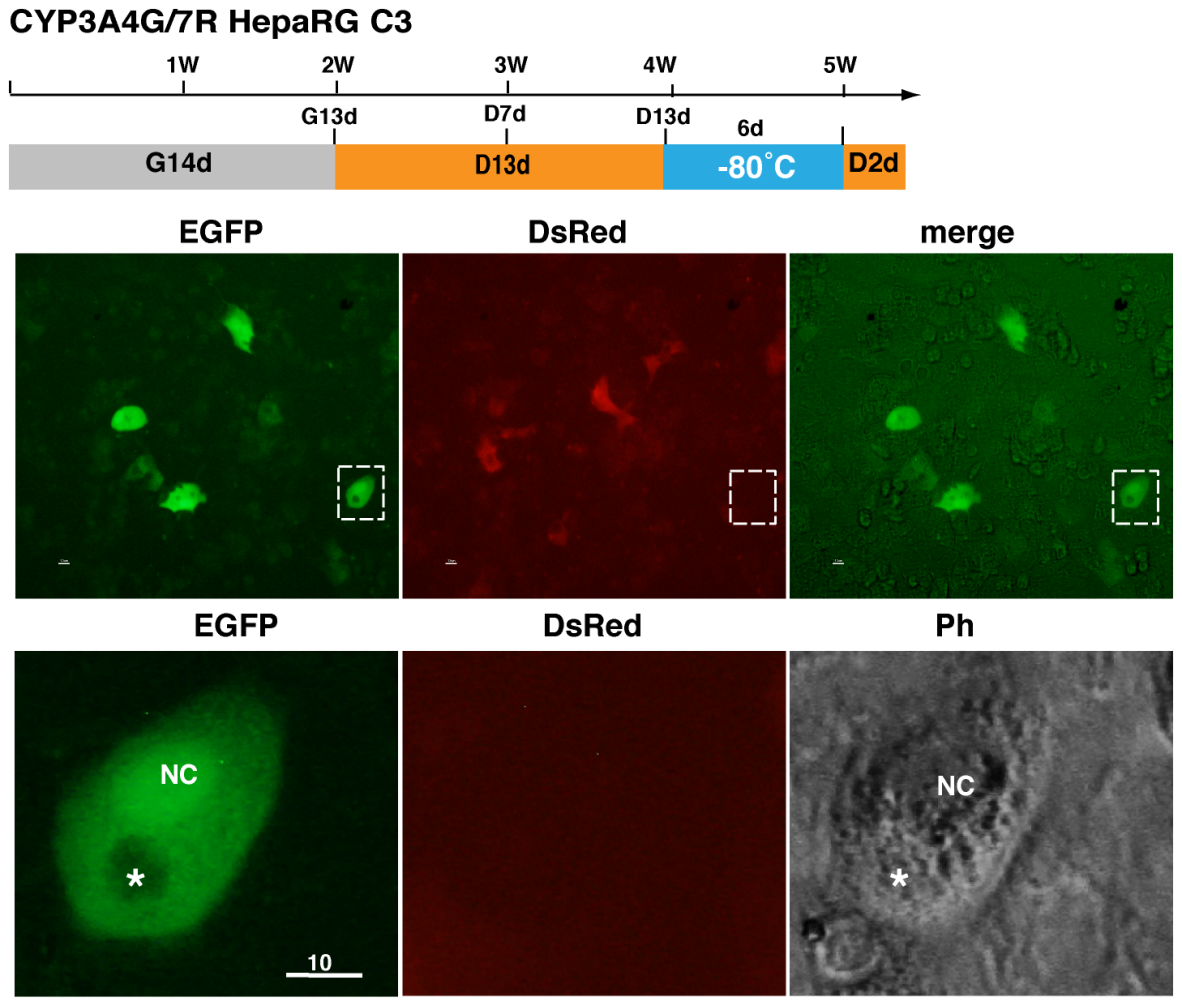
Recovery of frozen EGFP-positive HepaRG cells. HepaRG C3 cells at D13d were frozen period for 6 days at −80°C and then cultured for 2 days. Most cells were viable, including EGFP-positive cells. NC, nucleus; asterisk, an unknown large intracellular structure.

The HepaRG C3 cells at D13d were separated into single cells using trypsin and frozen at −80°C for 1 week in conventional freezing medium containing 10% DMSO. After being thawed, cells were cultured for 2 days. Many EGFP-positive cells were attached to the culture plate (Fig. 6). Of note, HepaRG cells often possessed a large intracellular structure, which was obviously different from the nucleus (Fig. 6, asterisk).

### CYP3A4 induction test in differentiated transgenic HepaRG cells

**Figure 7:**
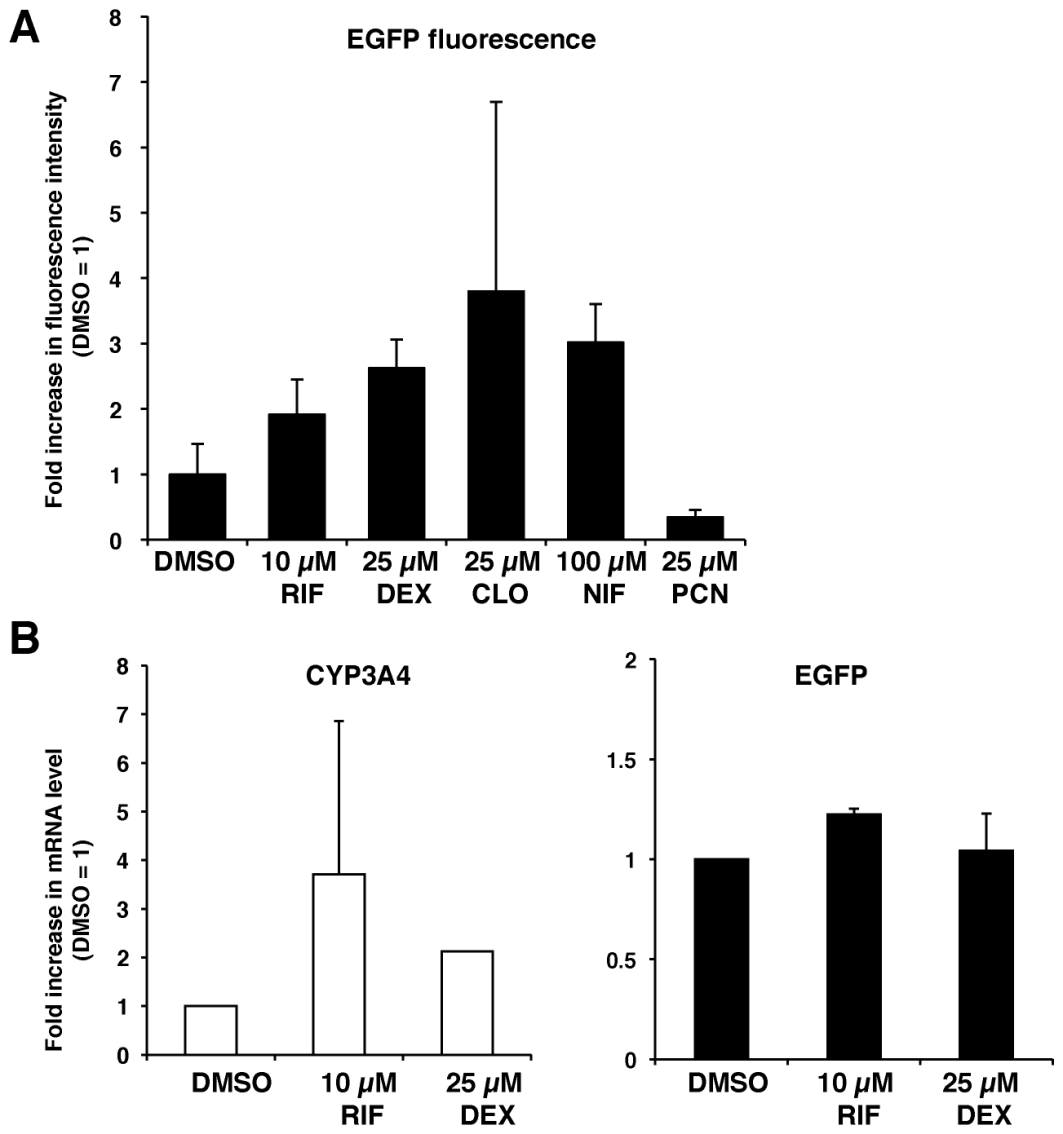
Induction tests of CYP3A4 transcription based on the fluorescence intensity of EGFP in transgenic HepaRG C3 cells. (A) CYP3A4 induction test. HepaRG C3 cells at D14d were prepared in three wells for each treatment with 10 µM RIF, 25 µM DEX, 25 µM CLO, 100 µM NIF, or 25 µM PCN for 48 h. The EGFP fluorescence intensity was measured in three areas per well. The mean ± SD of the fold increase relative to the level in 0.1% DMSO-treated cells (set at 1) is shown (n = 3). (B) HepaRG C3 cells at D14d were treated with 10 µM RIF or 25 µM DEX, and the mRNA levels of CYP3A4 and EGFP were determined by qRT-PCR. The mean ± SD of the fold increase in mRNA level relative to the level in 0.1% DMSO-treated cells (set at 1) is shown.

To ascertain whether the transgenic HepaRG C3 cells can be used to test CYP3A4 induction based on EGFP fluorescence intensity, the cells were grown on a 96-well plate and total fluorescence intensities were measured. HepaRG C3 cells at D14d were then treated with 10 µM RIF, 25 µM DEX, 25 µM clotrimazole (CLO), 100 µM nifedipine (NIF), 25 µM pregnenolone 16α-carbonitrile (PCN), or 0.1% DMSO for 48 h. The fold increases in the mean fluorescence intensities were calculated from triplicate samples, and the fold increase relative to the level in 0.1% DMSO-treated cells was determined (Fig. 7A). EGFP fluorescence intensity was 1.9±0.5-fold and 2.6±0.4-fold higher in cells treated with RIF or DEX than in DMSO-treated cells, respectively (p<0.05) (Fig. 7A). EGFP fluorescence intensity was 3.8±2.9-fold and 3.0±0.6-fold higher in cells treated with CLO or NIF than in DMSO-treated cells (p<0.05) (Fig. 7A). By contrast, PCN did not enhance EGFP fluorescence intensity (0.3±0.1-fold), consistent with the fact that PCN is an efficient inducer of Cyp3a in rats and mice, but not in humans (Fig. 7A). The mRNA levels of CYP3A4 and EGFP in these cells were analyzed. The mRNA level of CYP3A4 was 3.7±3.1-fold higher in the 10 µM RIF-treated cells than in DMSO-treated cells (p<0.05), but was only slightly increased in 25 µM DEX-treated cells (Fig. 7B, left). The mRNA level of EGFP was enhanced by RIF and DEX treatment, but this was not significant (Fig. 7B, right).

### Most EGFP-positive cells indirectly differentiate from DsRed-positive cells

**Figure 8:**
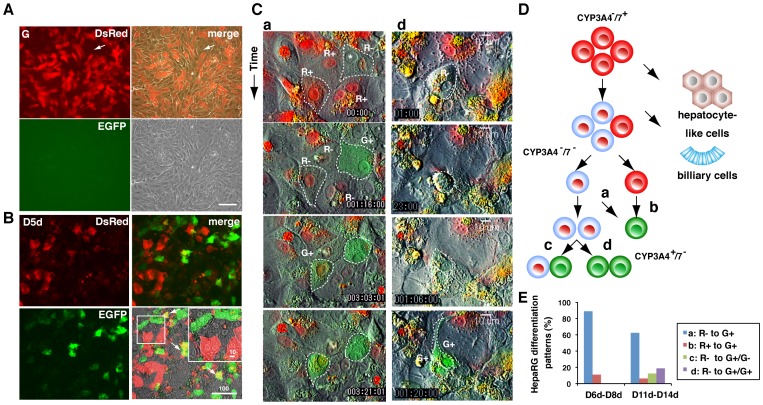
EGFP-positive cells do not always directly differentiate from DsRed-positive cells. (A) DsRed, EGFP, phase contrast, and merged images of transgenic HepaRG cells at the growing period (G). Undifferentiated transgenic HepaRG cells are blastic and DsRed-positive. Arrow indicates DsRed-negative cells. The size of the scale bar, 100 µm. (B) DsRed, EGFP, phase contrast, and merged images of transgenic HepaRG cells at D5d. EGFP-positive cells appear after most DsRed-positive cells have disappeared. Double-positive cells are rarely seen at D5d. Arrow indicates an exceptional double-positive cell. The size of the scale bar, 100 µm. Asterisk indicates a degenerated intracellular structure. (C) Sequential dual-color images from movies of HepaRG C3 cell differentiation between D6d and D14d. (a) A DsRed-positive cell (R+) becomes non-fluorescent (R-) and then EGFP-positive (G+); (d) a non-fluorescent cell divides into two cells, both of which are EGFP-positive. (D) A model for the course of hepatic cell differentiation from CYP3A7-expressing hepatoblastic precursors. Following differentiation of transgenic HepaRG cells, there is a colorimetric shift from red to green, which is separated by a period in which cells are not fluorescent without (a) or with (c and d) cell division. (E) Frequencies of EGFP-positive cells that differentiated through process a, b, c, or d between D6d and D8d or between D11d and D14d.

HepaRG C3 cells were used to resolve the differentiation process of HepaRG cells, which is similar to the differentiation of CYP3A7-expressing hepatoblasts into CYP3A4-expressing hepatocytes. HepaRG cells are bipotential cells and can differentiate into one of at least three types of cells, namely, CYP3A4-positive hepatocytes, CYP3A4-negative hepatic cells, and biliary-like cells as shown by immunostaining of HepaRG WT cells (Fig. S1). During the growing period, most cells were DsRed-positive, but some were negative for both DsRed and EGFP (Fig. 8A, arrow). After differentiation, DsRed was initially undetectable and then EGFP-positive cells appeared. During the course of differentiation, cells co-expressing DsRed and EGFP were only rarely observed (Fig. 8B, arrow). Thus, most CYP3A7-positive HepaRG cells are likely first phenotypically changed into hepatic cells or hepatic progenitors in which CYP3A7 expression is weak or absent. The differentiation of HepaRG C3 cells was monitored by the change in fluorescence from red to green in a movie captured over 96 h by live imaging microscopy (Fig. 8C and Movies S1–S4). As shown in Figure 8D, EGFP-positive cells appeared in at least four ways: (1) DsRed-positive cells first became non-fluorescent and then became EGFP-positive (pattern a in Fig. 8C and D, and Movie S1); (2) DsRed-positive cells directly became EGFP-positive (pattern b in Fig. 8D and Movie S2); (3) a non-fluorescent cell divided into two cells, one of which became EGFP-positive (pattern c in Fig. 8D and Movie S3); and (4) a non-fluorescent cell divided into two cells, both of which became EGFP-positive (pattern d in Fig. 8C and D, and Movie S4). As summarized in Figure 8E, the number of DsRed-positive cells decreased from D6d to D8d, and the division of non-fluorescent cells that gave rise to EGFP-positive cells was prominent between D11d and D14d. Thus, it can be concluded that most EGFP-positive cells were derived from DsRed-negative cells that possessed the ability to proliferate. Our dual-color live imaging system suggests that most CYP3A4-positive hepatocytes indirectly appear from CYP3A7-positive hepatoblastic cells.

## Discussion

In this study, we created transgenic HepG2 and HepaRG cells for real-time monitoring of CYP3A4 and CYP3A7 expression. These cells showed the following properties: (1) undifferentiated HepaRG cells were positive for DsRed fluorescence, whose expression was under the control of the enhancer and promoter regions of CYP3A7, (2) most cells became DsRed fluorescence-negative during the course of differentiation, and (3) mononuclear hepatocyte-like cells were strongly positive for EGFP, whose expression was under the control of the enhancer and promoter regions of CYP3A4. On the basis of this, HCS using these transgenic HepaRG cells might be useful to identify factors involved in the specialization of CYP3A4-expressing cells. Furthermore, EGFP fluorescence can be used to isolate functional HepaRG cells by FACS. These transgenic HepaRG and HepG2 cells can be used for CYP induction tests. However, caution should be exercised when using CYP3A4G/7R transgenic cells. In transgenic cells carrying reporter genes, the reporter can only be visualized when its mRNA expression is above a threshold level in each cell. Thus, the absence of fluorescence does not always indicate that the gene is not expressed at the mRNA level.

Xenobiotics induce CYP3A4 when they form a complex with PXR/RXR. This complex binds to the predominant cis-acting elements XREM and pNR, which are responsible for xenobiotic induction and are located within 8 kb upstream of the transcription initiation site [7]. The constitutive androstane receptor has a similar DNA-binding preference to PXR [12]. CYP3A4 and CYP3A7 exhibit a high degree of sequence identity at the protein level and within their XREM regions [9]. However, PXR is not detected in human fetuses, and CYP3A7 is mainly induced through a homodimer of the glucocorticoid receptor [10]. CYP3A4 and CYP3A7 differ in their capacity to perform monooxygenase reactions, as well as in their substrate specificities in such reactions [24]. Thus, human adult hepatocytes expressing CYP3A4, but not CYP3A7, are necessary for early-phase drug discovery. Moreover, transcriptional regulation of CYP3A genes appears to be species specific. Most notably, DEX induces the rat, rabbit, and human CYP3A genes, whereas PCN is an efficient CYP3A inducer in rats, but not in humans or rabbits. By contrast, RIF is a CYP3A4 inducer in humans and rabbits, but not in rats, mainly due to inter-specific differences in PXR molecules and the binding sequences of their targets [19], [20]. Mouse and human PXRs share only 75% identity in the amino acid sequences of their C-terminal ligand-binding domains, while their DNA-binding domains share 96% identity, which leads to the induction of human CYP3A4 by RIF, but not by PCN [25]. However, primary human hepatocytes cannot be used in a routine HCS-based system because they exhibit heterogeneity, many of their functions are poorly retained in culture, and they cannot be cultured in sufficient quantities. Therefore, in this study, we produced transgenic CYP3A4-expressing human hepatocyte-like cells in which CYP3A4 expression could be accurately monitored using reporter genes. The transgenic HepaRG cells are human hepatocyte-like cells in which the expression of EGFP and DsRed are under the control of the 35 kb enhancer/promoter region of CYP3A4 and the 23 kb enhancer/promoter region of CYP3A7, respectively, and in which all the regulatory elements are present. Therefore, these cells can be used to accurately monitor the expression levels of CYP3A4 and CYP3A7, and can be used in HCS assays.

Next, the advantages of the transgenic HepaRG and HepG2 cells created in this study are summarised and compared. HepG2 cells express low levels of CYP3A4; however, they can be cultured in large quantities, uniformly developed, and are inexpensive to culture. Moreover, EGFP expression was markedly induced in RIF-treated transgenic HepG2 cells. The CYP3A4 induction test can be completed within 3–5 days after transgenic HepG2 cells are seeded onto culture plates. By contrast, transgenic HepaRG cells require additional time for differentiation culturing, meaning a CYP3A4 induction test will take 4 weeks using these cells; however, this can be shortened by preparing frozen stocks of EGFP-positive HepaRG cells. Such cells are guaranteed to be CYP3A4-expressing hepatocytes, and their metabolic activity means they can be used for further analyses. Thus, the transgenic HepaRG cells provide a consistent and systematic assay system that can be used to generate reproducible data during drug discovery and development up to the preclinical stage. In this regard, HepaRG cells have more advantages than HepG2 cells.

To conclude, this 4G/7R BAC reporter system has advantages over other reporter systems. In assays using β-gal and luc [11], [12], [13], cell extracts must be prepared for enzymatic reactions. In the case of qRT-PCR analyses, further steps are required. Moreover, these assays measure CYP3A4 expression at one time-point only after chemical treatment, and thus there is a risk that the peak of CYP3A4 induction will be missed, leading to false-negative results. Furthermore, these systems require the cell number to be normalised among samples. By contrast, in this 4G/7R BAC reporter system, the total fluorescence intensities of EGFP and DsRed can be continuously measured, and rates of induction can be easily calculated from the values obtained before and after chemical treatments in each well. Thus, the cell number does not need to be normalised among samples. Changes in EGFP fluorescence intensity in these cells reflect changes in the mRNA level of endogenous CYP3A4. Taking all these factors into consideration, these transgenic cells substantially shorten or even eliminate many of the time-consuming steps required to estimate mRNA and protein levels during CYP3A4 induction tests. Differentiated HepaRG cells are a mixed cell population, in which the majority of cells have a different phenotype from CYP3A4-expressing hepatocytes. Therefore, most importantly, EGFP-positive HepaRG cells can be used instead of human hepatocytes in various analyses of CYP3A4-dependent drug metabolism, drug-drug interactions, hepatic toxicity, and the carcinogenicities of foreign substances.

We have generated donor cells containing a mouse chromosome vector in which a single 4G/7R BAC has been inserted using the Cre-loxP system. We are currently attempting to introduce this 4G/7R BAC into human induced pluripotent stem cells and Hprt-deficient mouse embryonic stem cells as an extra chromosome using microcell fusion. If this is achieved, there will be no need to generate loxP-bearing host cells to introduce a single BAC vector into a cell. Human pluripotent stem cells bearing the 4G/7R BAC will be useful for HCS of factors involved in the specification of CYP3A4-expressing cells. Such information will help produce functional hepatocytes for use in regenerative medicine.

## Materials and Methods

### Vector construction

To introduce two reporter genes into the BAC clone (RP11-757A13, Invitrogen), two vectors containing 200–500 bp arms homologous to the BAC sequences were generated. First, we created the LA-EGFP-pA-RA-CYP3A4 KI vector, which contained a XhoI linker, the left arm (200 bp promoter region of CYP3A4 immediately before the first ATG codon), the protein-coding sequence of EGFP, the Amp^r^ gene, the right arm (500 bp 3′-UTR of CYP3A4 immediately after the stop codon), and another XhoI linker. These were synthesised in this order by Integrated DNA Technologies, Inc. Second, the LA-DsRed-pA-RA-CYP3A7 KI vector was created, which was generated by using genomic PCR to synthesise a BglII linker, the left arm (the 200 bp promoter region of CYP3A7), and a SalI linker. The BglII-SalI fragment was inserted between SalI and BsaI within the multiple-cloning site of the pDsRed-Express-1 vector (BD Biosciences Clontech), which contains the protein-coding sequence of DsRed and the Kan/Neo^r^ gene. DNA fragments including a SmaI-XhoI linker, the right arm (200 bp 3′-UTR of CYP3A7 immediately after the stop codon), and a SmaI linker were synthesized by genomic PCR. The SmaI fragment was inserted into the BsaI site of pDsRed-Express-1. A loxP site was introduced into the BAC vector using the modification cassette vector II [26].

### BAC recombineering

The RP11-757A13 BAC clone was transferred into DY380 cells, a previously described E. coli strain (here, kindly provided by Dr. Stephen P. Creekmore of the National Cancer Institute, USA) that has been engineered for homologous recombination and gap-repair potencies [27]. The 4G/7R BAC vector was constructed *via* homologous recombination as described earlier. Briefly, 1 µg of BAC DNA was introduced into cells using a BioRad gene pulser set at 1.75 kV and 25 µF, and then Cm^r^ DY380 colonies were selected. A loxP site was introduced into the BAC through electroporation of the SacII-NdeI DNA fragments of the modification cassette II vector, and recombinants were isolated from Zeo^r^ colonies. Next, XhoI DNA fragments of LA-EGFP-pA-RA-CYP3A4 KI were introduced, and recombinant loxP-bearing BAC clones were isolated from Amp^r^/Zeo^r^ colonies. Finally BglII-XhoI DNA fragments of LA-DsRed-pA-RA-CYP3A7 KI were introduced, and recombinant BAC clones were isolated from Kan^r^/Amp^r^/Zeo^r^ colonies. Recombination was confirmed in each step *via* genomic PCR.

### Chemicals

Amp, Kan, CLO, NIF, DEX, DMSO, RIF, and PCN were purchased from Sigma-Aldrich Co. G418, and Zeo were purchased from Calbiochem and Life Technologies, respectively. Hyg and Cm were purchased from Wako. All reagents used in the CYP induction tests were dissolved in 0.1% DMSO.

### Cell culture and chemical treatment

HepaRG cells (Biopredic International) were maintained as previously described [16]. Briefly, HepaRG cells were seeded at a density of 2×10^4^ cells/cm^2^ on a culture dish, cultured in 710 growth medium to generate transgenic cells. HepaRG WT and C3 cells were expanded in 710 growth medium for 2 weeks on collagen-coated plastic or glass bottom plates, and then differentiated in 720 differentiation medium for up to 2 weeks in 5% CO_2_ at 37°C. The medium was replaced every 3 days. Transgenic HepG2 cells were seeded at a density of 0.3–1×10^5^ cells/cm^2^ on collagen-coated culture dishes in Dulbecco's modified Eagle's medium (Wako) supplemented with 10% (v/v) fetal bovine serum (Biowest), and then cultured for 24–48 h. Cells were treated with CYP inducers or 0.1% DMSO for 24 h for qRT-PCR analyses and for 48 h for fluorescence imaging analyses.

### Generation of transgenic cells

A loxP site was introduced into HepG2 and HepaRG cells using the 5′ HPRT-loxP/Pgk-hyg plasmid vector, which contains a loxP site and a Hyg-resistance gene, as previously described [28]. The NotI-linearized loxP vector was transfected using Lipofectamine LTX (Life Technologies). After 24 h, cells were dissociated and cultured in medium containing 400 µg/ml of Hyg for 7 days. The loxP-bearing transgenic clones were identified by genomic PCR and FISH, as described below. Then, 2 µg of purified CYP3A4G/7R BAC DNA was co-lipofected with 1 µg of the Cre expression vector pCAG-Cre. Transgenic clones, which were G418^r^ owing to the pSV40-neo gene derived from the pDsRed-Express-1 vector, were selected by culture in medium containing 800 µg/ml of G418. G418^r^ clones were picked, expanded, and characterised by genomic PCR.

### RNA extraction and qRT-PCR analyses

Total RNA was extracted from cells using the RNeasy Mini Kit (Qiagen), and cDNA was generated from 0.5 µg of total RNA using the Superscript III First-Strand Synthesis Kit (Life Technologies). cDNA derived from 10 ng of RNA was amplified in a 25 µl reaction using the Power SYBR Green PCR Master Mix Kit (Applied Biosystems) and a LightCycler 480 (Roche Applied Science). Primer sets used for qRT-PCR are described in Table 3.

**Table 3:** PCR primers used for gene expression analyses.

Sets	Genes	Size	Primers	Sequences (5′ to 3′)
1	CYP3A4 (NM_017460)	86 bp	hqCYP3A4-3A7-F	TTCATCCAATGGACTGCATAAAT
			hqCYP3A4-R	TCCCAAGTATAACACTCTACACAGACAA
2	hCYP3A7 (NM_000765)	86 bp	hqCYP3A4-3A7-F	TTCATCCAATGTGCTGCATAAAT
			hqhCYP3A7-R	TACCAAGTATAACACTCTATACAGACCA
3	EGFP	101 bp	GFP101-F	CTTCAAGGAGGACGGCAACA
			GFP101-R	CCTTGATGCCGTTCTTCTGC
4	DsRed	67 bp	DsRed q-RT F	GAAGGGCGAGATCCACAAG
			DsRed q-RT R	GGACTTGAACTCCACCAGGTA
5	ACTB (NM_001101)	76 bp	hβACTIN76-F	ATTGGCAATGAGCGGTTC
			hβACTIN76-R	GGATGCCACAGGACTCCAT

### Genomic PCR analyses

Genomic DNA was extracted from cultured cells using the Gentra Puregene Cell Kit (Qiagen), and PCR was performed in 20 µl of TaKaRa Ex Taq mixture (TaKaRa Bio) using the GeneAmp PCR system 9700 (Applied Biosystems). Primer sets used for genomic PCR analysis are summarised in Table 1.

### Imaging and quantification of fluorescence

Fluorescence images of transgenic HepG2 and HepaRG cells were acquired using a BZ-9000 fluorescence microscope (Keyence), a LEAP cell-processing workstation (Cyntellect), IncuCyte ZOOM (Essen BioScience), or VivaView FL (LCV-110) incubator fluorescence microscope (OLYMPUS). In each system, software to measure fluorescence intensities in the selected area was used in accordance with the manufacturer's protocol. For FACS analyses, HepG2 C87 cells were seeded at a density of 1×10^6^ cells on 60 mm culture dishes 2 days before use. HepaRG C3 cells at D14d were also prepared. After incubation for 48 h, cells were treated with DEX or RIF for 48 h. Cells were suspended in PBS, and the frequencies of EGFP- and/or DsRed-positive cells were immediately analyzed or sorted using a FACSAria flow cytometer (BD Biosciences).

### Chromosome preparation and FISH analyses

Cells were treated with 0.3 µg/ml colcemide (Demecolcine, Sigma-Aldrich) for 30 min, incubated for 15 min in 0.075 M KCl, and then gradually fixed with a mixture of methanol and acetic acid (3∶1). Chromosome spreads were prepared on glass slides using the air-drying method. DNA of the loxP-bearing and BAC vectors were labelled with biotin-UTP and dig-UTP, respectively, using a standard nick translation kit (Roche Applied Science). Chromosomal DNA and labelled probes were denatured and hybridized, and hybridised signals were detected using streptavidin-FITC and anti-dig-Rhodamine, as previously described [29]. Chromosomal DNA was counterstained with DAPI (Sigma-Aldrich). A human mFISH probe was purchased from MetaSystems, and mFISH was performed in accordance with the manufacturer's procedure. FISH signals were detected using an Axio Imager-Z2 microscope (Carl Zeiss).

### Immunohistochemical analyses

HepaRG cells were fixed with PBS containing 2% (w/v) paraformaldehyde for 10 min, washed three times (5 min each) in PBS, and permeabilized with 0.1% Triton X-100 for 10 min. After washing with PBS again, cells were treated with PBS containing 2% skimmed milk for 30 min at room temperature, and then with primary antibodies diluted in PBS containing 2% skimmed milk for 1 h at room temperature. The primary antibodies used were rabbit polyclonal anti-human cytochrome P450 3A4 (1∶500, Enzo), mouse monoclonal anti-CK8/18 (1∶500, PROGEN Biotechnik), rabbit polyclonal anti-human AAT (1∶100, DakoCytomation), mouse monoclonal anti-human ALB antibody (1∶500, Sigma-Aldrich), and/or mouse monoclonal anti-human CK19 (1∶500, DakoCytomation). Secondary antibodies used were Alexa488-conjugated mouse IgG and Alexa546-conjugated rabbit IgG (1∶500, Molecular probes, Life Technologies). Samples were washed three times in PBS containing 0.05% Tween-20. Finally, nuclei were stained with Prolong Gold antifade reagent containing DAPI (Life Technologies).

### Western blot analyses

HepaRG WT and C3 cells at D7d, and C87 HepG2 cells were suspended in RIPA buffer (Sigma-Aldrich) and placed on a rotator for 30 min at 4°C. Proteins were collected by centrifugation at 12,000 rpm for 20 min at 4°C. Thereafter, 10–20 µg of protein mixed with 25% LDS sample buffer (NuPAGE) was separated by electrophoresis on a 5–20% SDS-polyacrylamide gradient gels (ATTO) and transferred to a polyvinylidene fluoride membrane. For each of sample, three western blots were prepared. Next, the membranes were pre-hybridized with PBS containing 3% skimmed milk (Difco, BD) overnight at 4°C, and then incubated with the primary antibody diluted in PBS containing 3% skimmed milk for 1 h at room temperature. First, the primary anti-CYP3A4/7antibody (rabbit polyclonal, 1∶500; Enzo) was used. The hybridized bands were visualized using the ECL Plus Western Blotting Detection Kit (GE Healthcare). Second, the membrane was co-incubated with two primary antibodies, namely, anti-EGFP (mouse monoclonal, 1∶200; Santa Cruz Biotechnology, Inc.) and anti-β-actin (mouse monoclonal, 1∶5,000; Abcam). The relative expression levels of CYP3A4/7 and EGFP in cells were calculated by normalizing the intensities of these bands to that of β-actin using an LAS-3000 imaging system (Fujifilm, Tokyo, Japan).

### Statistical analyses

The sample size was limited and it was unclear whether the data were normally distributed; therefore, the Mann-Whitney U-test was used. We used p-values obtained from the Z score, which describes where a value is located in the distribution. |Z|  = 0 is at the center of the distribution, whereas a Z score of 2.0 or above is located in the tails of the distribution. A 95% range (p<0.05) corresponds to a Z score of <−1.96 or >+1.96. When the absolute value of the calculated Z score was between 1.96 and 1.65, the results were considered a tendency (p<0.1).

## Supporting Information

Figure S1 **CYP3A4-expressing cells appear following the differentiation of HepaRG cells.** (A) Strategy for hepatic differentiation of HepaRG cells *in vitro* and the morphologies of cells at G14d (left) and D14d (right). The size of the scale bar is 100 µm. (B) Immunostaining of HepaRG cells for (a) CYP3A4/7 and CK8/18, (b) AAT and CK19, or (c) CYP3A4/7 and ALB at D14d. The size of the scale bar is 100 µm. (C) qRT-PCR analyses showing the mRNA levels of CYP3A4 and CYP3A7 in HepaRG cells from G14d to D15d. The mean values of three independent measurements were calculated, and the mRNA level relative to the level in AL or FL (set at 100) is shown. (D) CYP3A4 transcription induction test. HepaRG WT cells were treated with 10 µM RIF or 25 µM DEX, and the mRNA levels of CYP3A4 and CYP3A7 were determined by qRT-PCR at D14d. The mean values of three independent measurements were calculated, and the fold increase relative to the level in 0.1% DMSO-treated cells (set at 1) is shown.
(TIF)

Movie S1 **DsRed-positive cells become DsRed-negative and then EGFP-positive (pattern a in** **Fig. 8C and D** **).**
(AVI)

Movie S2 **A DsRed-positive cell directly becomes EGFP-positive (pattern b in** **Fig. 8D** **).**
(AVI)

Movie S3 **A DsRed-negative cell divides to generate an EGFP-positive cell (pattern c in** **Fig. 8D** **).**
(AVI)

Movie S4 **A DsRed-negative cell divides to generate two EGFP-positive cells (pattern d in** **Fig. 8C and D** **).**
(AVI)
