# Supplementary figures and images for: Dual-Color Fluorescence Imaging to Monitor CYP3A4 and CYP3A7 Expression in Human Hepatic Carcinoma HepG2 and HepaRG Cells
Source: PLoS One. 2014 Aug 7;9(8):e104123. doi: 10.1371/journal.pone.0104123 (PMC4125183; doi:10.1371/journal.pone.0104123)

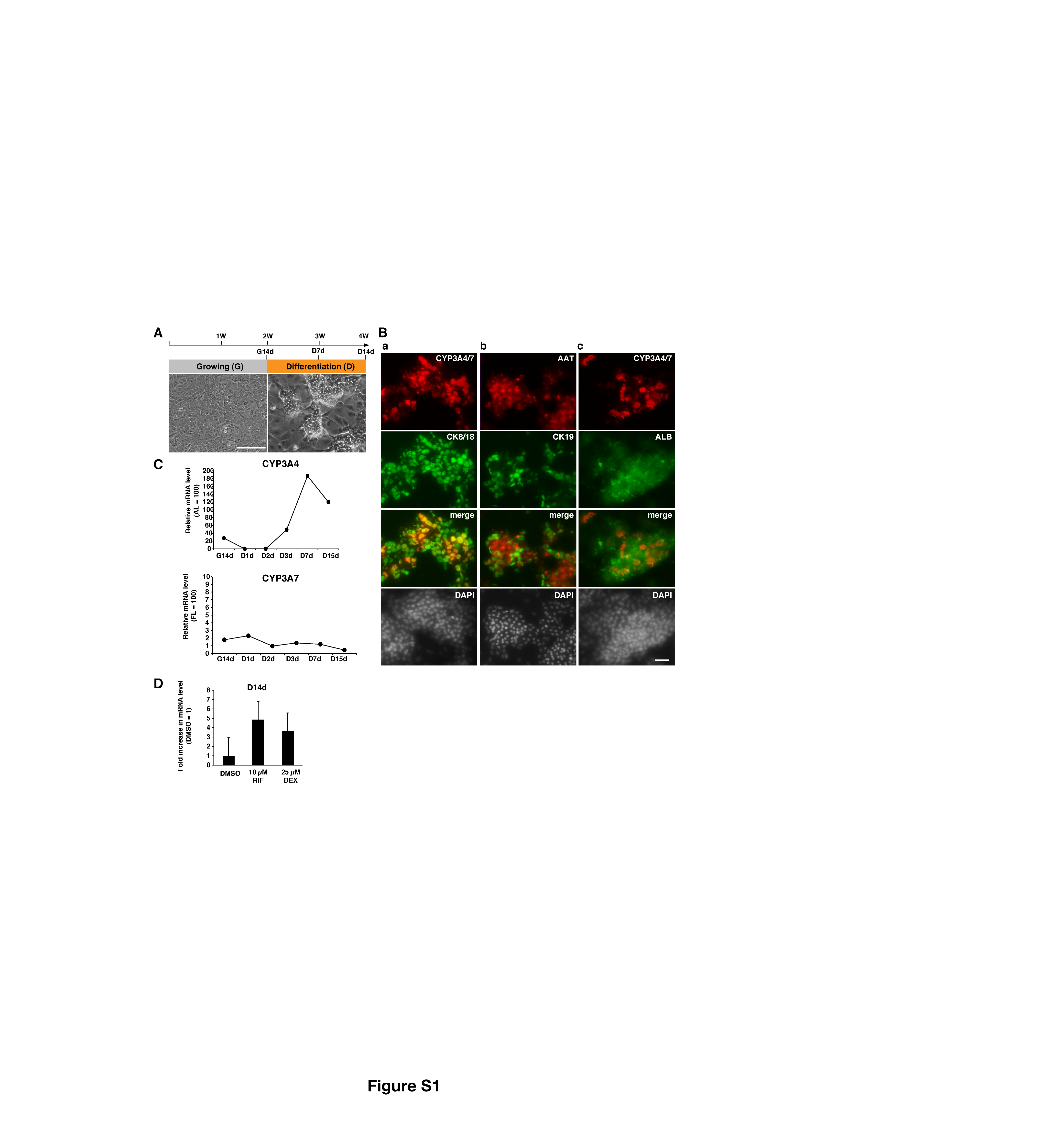

Supplement: Figure S1 — CYP3A4-expressing cells appear following the differentiation of HepaRG cells. (A) Strategy for hepatic differentiation of HepaRG cells in vitro and the morphologies of cells at G14d (left) and D14d (right). The size of the scale bar is 100 µm. (B) Immunostaining of HepaRG cells for (a) CYP3A4/7 and CK8/18, (b) AAT and CK19, or (c) CYP3A4/7 and ALB at D14d. The size of the scale bar is 100 µm. (C) qRT-PCR analyses showing the mRNA levels of CYP3A4 and CYP3A7 in HepaRG cells from G14d to D15d. The mean values of three independent measurements were calculated, and the mRNA level relative to the level in AL or FL (set at 100) is shown. (D) CYP3A4 transcription induction test. HepaRG WT cells were treated with 10 µM RIF or 25 µM DEX, and the mRNA levels of CYP3A4 and CYP3A7 were determined by qRT-PCR at D14d. The mean values of three independent measurements were calculated, and the fold increase relative to the level in 0.1% DMSO-treated cells (set at 1) is shown. (TIF) [file pone.0104123.s001.tif]
